# The Impact of Different Sources of Fluctuations on Mutual Information in Biochemical Networks

**DOI:** 10.1371/journal.pcbi.1004462

**Published:** 2015-10-20

**Authors:** Michael Chevalier, Ophelia Venturelli, Hana El-Samad

**Affiliations:** 1 Department of Biochemistry and Biophysics, California Institute for Quantitative Biosciences, University of California San Francisco, San Francisco, California, United States of America; 2 Department of Bioengineering, University of California, Berkeley, Berkeley, California, United States of America; Johns Hopkins University, UNITED STATES

## Abstract

Stochastic fluctuations in signaling and gene expression limit the ability of cells to sense the state of their environment, transfer this information along cellular pathways, and respond to it with high precision. Mutual information is now often used to quantify the fidelity with which information is transmitted along a cellular pathway. Mutual information calculations from experimental data have mostly generated low values, suggesting that cells might have relatively low signal transmission fidelity. In this work, we demonstrate that mutual information calculations might be artificially lowered by cell-to-cell variability in both initial conditions and slowly fluctuating global factors across the population. We carry out our analysis computationally using a simple signaling pathway and demonstrate that in the presence of slow global fluctuations, every cell might have its own high information transmission capacity but that population averaging underestimates this value. We also construct a simple synthetic transcriptional network and demonstrate using experimental measurements coupled to computational modeling that its operation is dominated by slow global variability, and hence that its mutual information is underestimated by a population averaged calculation.

## Author Summary

This work demonstrates how different sources of variability within biochemical networks impact the interpretation of information transmission. These sources are the intrinsic noise generated within the pathway of a single cell, variability due to initial conditions and/or global parameters across the population. A theoretical analysis of a simple signaling pathway and experimental exploration of a synthetic circuit are used to discuss the contributions of these sources of variability to information transmission using mutual information as a metric.

## Introduction

To survive in challenging conditions, cells need to detect, transduce, and process signals from their environment. A cell’s ability to precisely process environmental signals is limited by intrinsic fluctuations and variability of its cellular processes. This variability takes root in the stochastic nature of biochemical reactions. For a given pathway, this includes the stochastic steps involved in transcription and translation [1–4] as well as diffusion-reactions, dissociations, allosteric changes, and degradation of biological molecules. A signal propagates across cellular networks through molecules undergoing these various reactions, and gets distorted and altered by their probabilistic nature. Therefore, metrics for quantifying the limits of faithful information propagation (signaling fidelity) in biological pathways are crucial for understanding their information processing and transduction capabilities.

Mutual information [5] (MI) is a natural metric for characterizing information transmission between the inputs of a stochastic network and its nodes. MI quantifies the level of precision with which a given node(s) in a network estimates and responds to an input(s) by accounting for both the mean and variability in the response. Recent studies have used MI to characterize information transmission between environmental inputs and transcription factors in a number of genetic circuits [6–10]. In these studies, steady-state MI was computed for a variety of *in silico* networks to assess their stationary response as a function of input dose. More recently, these ideas were extended to optimize time-dependent MI in delay circuits with binary inputs, and MI was used to discuss maximally informative network topologies in these contexts [11]. In addition, time dependent MI calculations were used to obtain fundamental limits on the suppression of molecular fluctuations for different network topologies [12].

Several experimental studies have also used MI to assess signaling fidelity. MI was used as a metric to argue that negative feedback enables dose-response alignment and enhances information transmission in the pheromone pathway in yeast [13]. Similarly, MI was used to estimate time-dependent information transfer in tumor necrosis factor (TNF) signaling, and to assess transmission bottlenecks in this system [14]. Recently, robustness and compensation of information transmission in different pathways and pharmacological perturbations were attempted in PC12 cells using similar measurements [15]. These experimental studies relied on driving isogenic cell populations with various inputs, and then calculating the mutual information based on the overall variability in the population response. Such calculations mostly found low MI values, suggesting that cellular pathways might have on average low information transmission capacity. In this work, we argue that these calculations often under-estimate MI of a pathway in a single cell, since they do not account for 1) *variability in initial conditions* and 2) *variability that is extrinsic to the pathway*. The overall effect of these two sources of variability is that any single cell has a quantitatively distinct input-output relationship [3, 4, 16] and that calculations that take this into account are needed for more accurate estimation of MI from experimental data. By assuming that extrinsic variability manifests as cell-to-cell differences in a global parameter, such as translation capacity, we demonstrate in a simple *in silico* circuit that mixing cells with different parameters sets (and/or different initial conditions) reduces the value of the computed MI. We also argue this point experimentally by building a simple synthetic circuit that exhibits strong extrinsic variability, and then demonstrating with the help of computational modeling that single cells within the population have a larger mutual information than that exhibited by the averaged population. These results indicate that cells might possess higher capacity for information transmission than previously appreciated.

## Results

**Fig 1:**
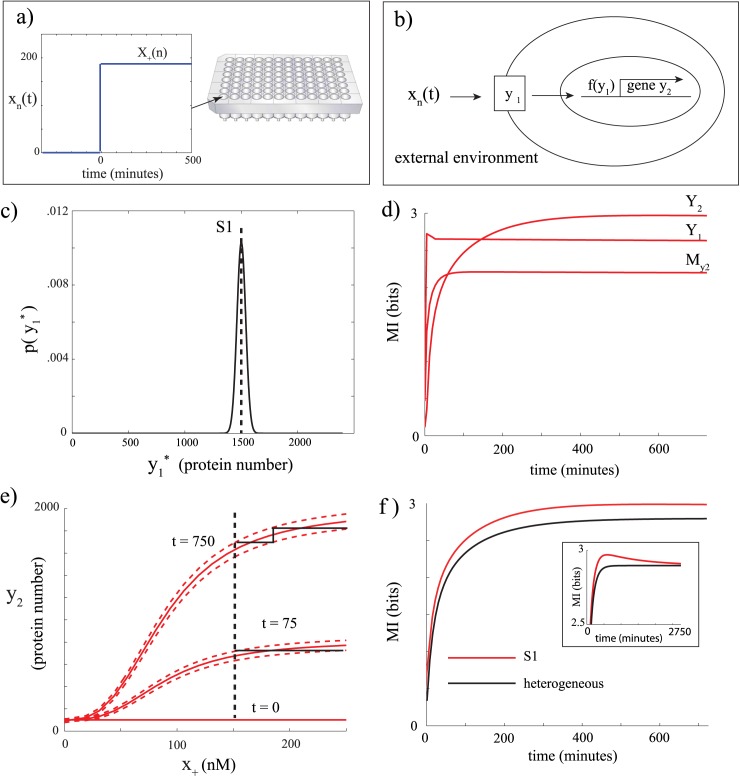
Time-dependent mutual information transmission in a simple biochemical circuit. a) Every well contains the same type of isogenic cell population where each well receives a different step input sampled from a uniform distribution. The resulting time series data is used to compute time-dependent mutual information. b) Schematic of pathway. c) Initial distribution of $Y_{1}^{*}$ before any input is given, reflecting cell-cell variability in initial conditions. In this case, a single parameter set is used and the distribution is the result of intrinsic variability of the circuit. S1 denotes the mean of the distribution. d) *I*(*x*_+_, *y*_1_, *t*∣*S*1), *I*(*x*_+_, *M*_*y*_2__, *t*∣*S*1), and *I*(*x*_+_, *y*_2_, *t*∣*S*1). e) Time-dependent dose-response relationships at *t* = 0, 75 and *t* = 750 minutes between *y*_2_ and the input. The vertical black dashed line is where *x*_+_ = 150 nM. f) Mutual information between *y*_2_ and the input computed for heterogenous initial conditions (full distribution in panel c, black) and for homogeneous initial conditions (S1, red). Inset: same data but with time plotted to 2750 minutes and the MI plotted between 2.5 to 3 bits.

To compute mutual information in a given biological network, we apply simple step functions [14] of the appropriate environmental input to *N* populations of the same isogenic cells. The step function is mathematically defined as *x*(*t*) = 0 for *t* < 0 and *x*(*t*) = *X*_+_ for *t* ≥ 0, where *X*_+_ is a constant within a given population. For each of the N populations, *X*_+_ is sampled from a discrete uniform distribution, *p*_*u*_(*x*_+_), over the range of interest (0 to *X*_*max*_). The uniform distribution represents an unbiased distribution (other than the choice of *X*_*max*_) that has been routinely applied to steady-state mutual information calculations [6]. Experimentally, one can implement this scheme by growing replicas of the same culture in an *N*-well plate and stimulating each well with a different step function as defined above (Fig 1a). For a given population *n* and sampled input amplitude *X*_+_(*n*), the stochastic time-dependent response of measurable proteins *y* = [*y*_1_ … *y*_*m*_] of the population at *t* will be *p*(*y*, *t*∣*X*_+_(*n*)). For a general *x*_+_ between 0 and *X*_*max*_, we interpolate the central moments of adjacent sampled distributions to construct *p*(*y*, *t*∣*x*_+_). The time-dependent mutual information is then given by

$$\begin{array}{ccc} I\left(x\left(t\right);y,t\right) & = & \sum_{x(t)}\sum_{y}p\left(y,t|x\left(t\right)\right)p\left(x\left(t\right)\right)\log_{2}\frac{p(y,t|x(t))}{p(y,t)}\\ & = & \sum_{x_{{}_{+}}}\sum_{y}p\left(y,t|x_{{}_{+}}\right)p_{u}\left(x_{{}_{+}}\right)\log_{2}\frac{p(y,t|x_{{}_{+}})}{p(y,t)}\\ & = & I(x_{{}_{+}};y,t) \end{array}$$ (1)

The value of *N* (that is the number of experiments) can be chosen based on well established methods [14] to approximate the MI in Eq (1) (see Materials and Methods for further details).

We first illustrate MI calculations using an *in silico* model of a simple signaling cascade (Fig 1b). Here the input *X*_*n*_(*t*) causes the transformation of the inactive molecule $Y_{1}^{*}$ to its active form *Y*_1_. *Y*_1_ could be a receptor or transcription factor responsive to the given input. *Y*_1_ in turn activates transcription of *Y*_2_, whose mRNA is denoted as *M*_*y*_2__ (chemical equations are detailed in Materials and Methods with the parameter values listed in Table 1). The uniform input distribution is between 0 nM and *X*_*max*_ = 250 nM. We found that for this system and its corresponding parameters, *N* ≥ 20 was a conservatively large number of experiments to approximate MI.

**Table 1:** Parameters for circuit in numerical example.

parameter:	$\beta_{y_{1}^{*}}$ (mol s^−1^)	$\gamma_{y_{1}^{*}}=\gamma_{y_{1}}$ (s^−1^)	*θ*_*x*_ (mol^−1^ s^−1^)	*θ*_*y*_1__ (s^−1^)	*n*_1_	*y*_1_0__ (mol)
value:	.01	.0000067	.001	1.5	3	85
parameter:	$\beta_{m_{2}}^{*}$ (mol s^−1^)	*β*_*m*_2__ (mol s^−1^)	*γ*_*m*_2__ (s^−1^)	*β*_*y*_2__ (s^−1^)	*γ*_*y*_2__ (s^−1^)
value:	.0083	.1583	.00083	.0014	.00014

### Contribution of initial condition variability to time-dependent mutual information

We first assumed that this circuit is isolated from the rest of the cell, and that any stochasticity it exhibits is only the result of its chemical reactions (intrinsic variability). When this system is unstimulated (*t* ≤ 0), its molecular species assume a joint steady-state distribution, $p(y_{1}^{*},y_{1},M_{y_{2}},y_{2},t\leq 0)$. As an example, we show the marginal distribution of $Y_{1}^{*}$ in Fig 1c. This distribution represents the range of initial conditions in $Y_{1}^{*}$ that a population containing this network would exhibit before any input is applied.

We will first compute the MI of the network while ignoring this initial distribution of states, assuming that all cells in the population start from the same initial condition (for state *S*1, this is the mean of the initial joint distribution, see Fig 1c) which we refer to as a homogeneous initial condition. This could be thought about as the mutual information of one cell in that population. We plot the time-dependent mutual information between the input and the different species of the circuit: *I*(*x*_+_; *y*_1_, *t*∣*S*1), *I*(*x*_+_; *M*_*y*_2__, *t*∣*S*1), *I*(*x*_+_; *y*_2_, *t*∣*S*1) (Fig 1d). The MI from the input to *y*_1_, *I*(*x*_+_; *y*_1_, *t*∣*S*1), has rapid dynamics, peaking initially and decaying with time to a steady-state. The initial peak in this MI is due solely to the activation and inactivation of *y*_1_, while the subsequent decrease to steady-state is due to the fluctuations in the synthesis and degradation of *y*_1_. By contrast, the MI from the input to *M*_*y*_2__ (*I*(*x*_+_; *M*_*y*_2__, *t*∣*S*1)) is slower and on the order of tens of minutes, while that of the protein *y*_2_ (*I*(*x*_+_; *y*_2_, *t*∣*S*1)) is on the order of hours. This is not unexpected, as the mutual information signals for each species follow the causality of the circuit where *y*_2_ shows the largest delay.

The increase of *I*(*x*_+_; *y*_2_, *t*∣*S*1) as a function of time has an intuitive explanation in terms of *y*_2_ dynamics. To visualize this, we plot ${\bar{y}}_{2}(n,t)$, the mean of *y*_2_ as well as ${\bar{y}}_{2}(n,t)\pm \sigma_{y_{2}}(n,t)$ versus *X*_+_(*n*) for *t* = 0, 75, and 750 minutes (Fig 1e, red lines), where *σ*_*y*_2__(*n*, *t*) is the standard deviation in *y*_2_. We will refer to these plots as the *time-dependent dose response relationships*. For *t* = 750, more values of *x*_+_ are resolvable from measurement of *y*_2_ than at time *t* = 75. For example, for *x*_+_ > 150, steps in the dose response curves constrained between the standard deviations (Fig 1e, black lines for t = 75 and 750 minutes) approximate how well a measurement in *y*_2_ can infer the value of *x*_+_. At time 750, about 2 steps are resolvable allowing for two distinct ranges of *x*_+_ to be inferred. While for *t* = 75 minutes, only one distinct range of *x*_+_ is inferrable. The larger the number of resolved states, the higher the value of the mutual information.

When mutual information is calculated between the input and a given node over the entire time duration of the signals, the mutual information between the input and each successive node has an upper bound equal to that of the prior node. This is known as the data processing inequality [5]. However, since we are evaluating the time-dependent MI at a given time *t*, the instantaneous value *y*_2_ can have more information about the input than *y*_1_. Indeed, at *t* approximately greater than 150 minutes, we find that the MI *I*(*x*_+_; *y*_2_, *t*∣*S*1) is greater than *I*(*x*_+_; *y*_1_, *t*∣*S*1) or *I*(*x*_+_; *M*_*y*_2__, *t*∣*S*1) (Fig 1d).This is because for the particular parameter set used in this example, the noise propagated from *y*_1_ onto *y*_2_ is averaged out, and the only variability in *y*_2_ stems from its own production and degradation. As a result, *I*(*x*_+_; *y*_2_, *t*∣*S*1) can be modulated to be higher or lower than *I*(*x*_+_; *y*_1_, *t*∣*S*1) by changing the rates of *y*_2_ production and degradation [17]. On the other hand, increasing the number of *y*_1_ molecules would increase its mutual information as this would reduce the noise in the *y*_1_ signal. Therefore, the mutual information at each node of this pathway can be modulated through choice of kinetic parameters. Similar observations that filtering can improve time-dependent MI between success nodes have been discussed in the context of other types of pathways [18].

Next, we examined mutual information while accounting for the fact that cells assume a distribution of initial states across the population upon receiving the input stimulus. We do so by incorporating the pre-stimulus steady state initial joint distribution into the MI calculations. This variability in initial conditions transiently reduces the MI (Fig 1f). At steady-state, the mutual information curves computed for a single or a distribution of initial states eventually converge onto each other at approximately *t* = 2750 minutes (Fig 1f, inset). This convergence at longer times occurs because a population in which every cell assumes the same exact initial conditions will eventually produce a heterogeneous distribution of states due to the intrinsic stochasticity of the biochemical reactions. For the values of parameters used in this example, the convergence of the two MI curves proceeds very slowly. Therefore, even when only intrinsic fluctuations are present, with no extrinsic contributions to variability, and for a given distribution of initial conditions, a single cell still transiently assumes, on average, a higher time-dependent mutual information than the whole population. In our case, this difference is very modest.

### Time-dependent mutual information transmission with global parameter variation

Thus far, in our MI calculations, we have only accounted for variability in initial conditions given a single parameter set for the pathway. More realistically, any given pathway in a cell is subjected to variability through coupling to other cellular activities. This is known as extrinsic noise to distinguish it from intrinsic noise generated by the pathway itself. There are many extrinsic sources of variability that cellular pathways experience. For example, different cells may contain different numbers of polymerases or ribosomes, and hence have different capacities for transcription and translation [3]. This extrinsic variability can be accounted for in many ways, the simplest is to assume that the transcription or translation rate constants themselves can assume different values in different cells across the population.

To demonstrate the contribution of extrinsic variability to MI calculations, we consider a simple case where cells in the population have different translation rates. To do so, we add a stochastic global variable, *G*, which affects the protein creation rates such that ${\hat{\beta }}_{y_{1}^{*}}=\beta_{y_{1}^{*}}G/\bar{G}$ and ${\hat{\beta }}_{y_{2}}=\beta_{y_{2}}G/\bar{G}$, where $\beta_{y_{1}^{*}}$ and *β*_*y*_2__ are the nominal values for the parameters used above. In this way, the protein creation rates keep their mean value, but fluctuate because of their coupling to *G*. For this example, *G* follows a memoryless birth/death process such that the mean of *G* is $\bar{G}=\beta_{g}/\gamma_{g}$ (*β*_*g*_, *γ*_*g*_ are the birth and death rates). It follows that *G* has a coefficient of variation given by $\eta_{g}=1/\sqrt{\bar{G}}$.

**Fig 2:**
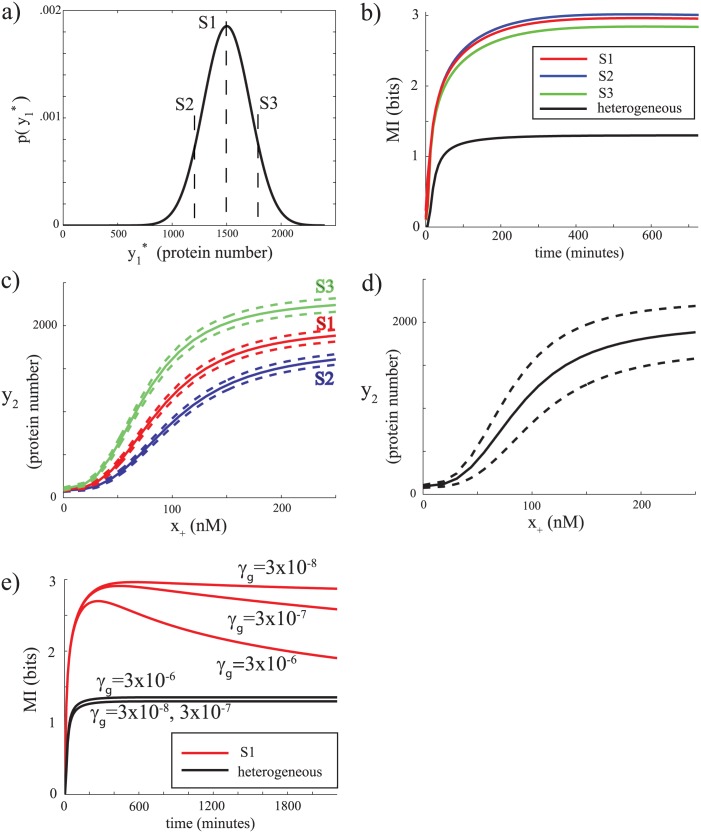
Global variability has large impact on mutual information. a) Initial distribution of $Y_{1}^{*}$ before any input is given, reflecting cell-cell variability in initial conditions. In this case, the protein synthesis parameter is stochastic, reflecting a globally varying source of noise. S1 denotes the mean of the distribution, while S2 and S3 denote cells that are one standard deviation away from the mean. b) Time-dependent mutual information computed for heterogeneous initial conditions (the whole distribution in panel a, black), or homogeneous initial conditions (S1: red, S2: blue, and S3: green) c) Time-dependent dose-response relationships between *y*_2_ and input at t = 750 minutes for a population starting from homogeneous initial conditions corresponding to *S*_1_, *S*_2_, and *S*_3_. d) Time-dependent dose-response relationship between *y*_2_ and input for heterogeneous initial conditions corresponding to full distribution in panel a. Calculations for panels a-d correspond to a slowly fluctuating global variable with *γ*_*g*_ = 3 × 10^−8^. e) Mutual information for different timescales of the fluctuations in the global variable.

First, setting $\bar{G}=50$, we chose *β*_*g*_ = 1.5 × 10^−6^ mol-s^−1^ and *γ*_*g*_ = 3 × 10^−8^ s^−1^. These values establish a stationary distribution of states, which we use as an initial distribution for the MI calculations. Fluctuations in the translation rate induce extra variability in the pathway components (compare the initial distributions of $Y_{1}^{*}$ in Fig 2a to Fig 1c). As a result, mutual information calculations with this added extrinsic variability (and using the population distribution of initial conditions) show that *I*(*x*_+_; *y*_2_, *t*) is now drastically reduced compared to the case when a single parameter set is used to represent the lack of global variability (compare black line in Fig 2b with value in Fig 1d). Here also, as expected, MI calculations from a single initial state corresponding to parameters $G=\bar{G}$ (state *S*1), $G=\bar{G}-\sqrt{\bar{G}}$ (state *S*2) and $G=\bar{G}+\sqrt{\bar{G}}$ (state *S*3), generate high transient values (red (*S*1), blue (*S*2) and green (*S*3) curves in Fig 2b). This discrepancy between single cell and population MI is further highlighted by examining the time-dependent dose response relationship between *y*_2_ and *x*_+_ at *t* = 750 (Fig 2d (full population) and Fig 2c (*S*1, *S*2, and *S*3). Again, the sub-populations generated from *S*1, *S*2, *S*3 each have little variability (high mutual information) relative to the full population.

While constraining $\bar{G}=50$, we investigated the time-dependent MI for different values of *β*_*g*_ and *γ*_*g*_. Our original choice of *γ*_*g*_ = 3 × 10^−8^ forces *G*, and hence the translation rates ${\hat{\beta }}_{y_{1}^{*}}$ and ${\hat{\beta }}_{y_{2}}$, to fluctuate very slowly. Therefore, the convergence of the MI values computed from a single initial condition versus the full distribution also proceeds slowly. As *γ*_*g*_ increases, this convergence proceeds faster (Fig 2e). Therefore, these results indicate that the mutual information of a pathway can be severely underestimated by population-based measurements if the pathway is subjected to global fluctuations that proceed on a slower timescale than the pathway itself.

### Probing the mutual information of a simple synthetic circuit

**Fig 3:**
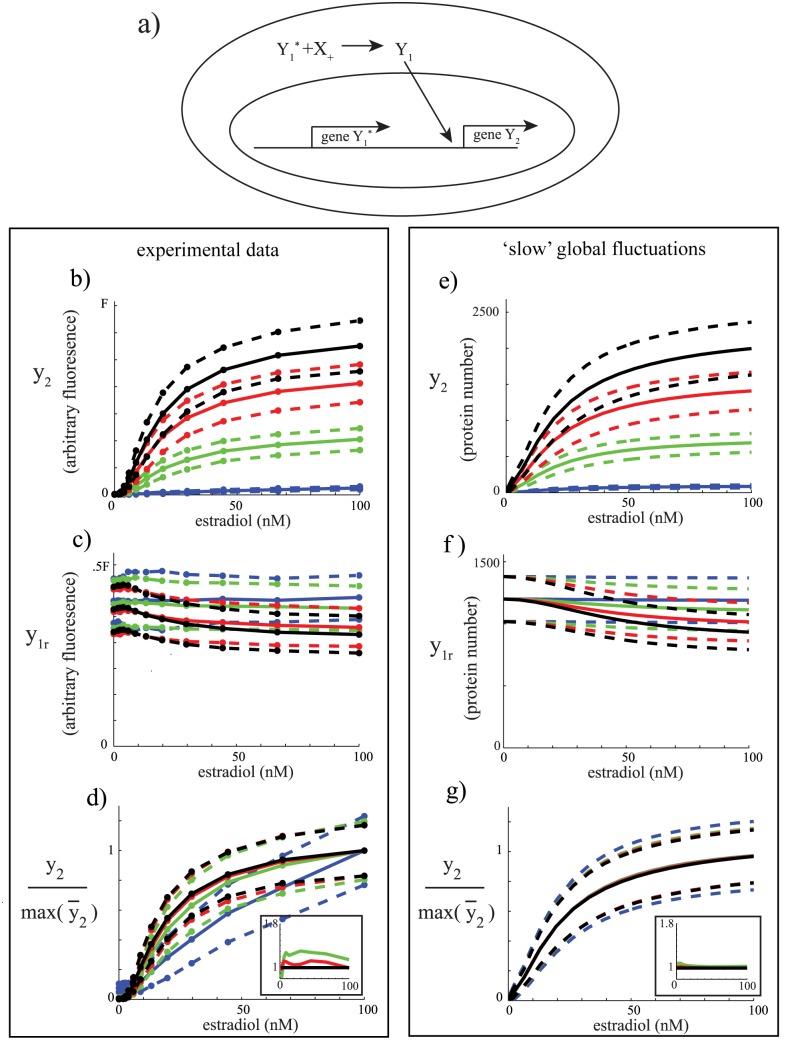
Variability in a transcriptional synthetic circuit is dominated by slowly fluctuating global variable. a) Schematic of the simple synthetic circuit. b-g) Dose-response relationships for *t* = 65 (blue), 165 (green), 330 (red), and 580 (black) minutes. Insets in d and g represent the ratio of the noise at time *t* to that at time *t* = 580 where for green: *t* = 165, red: *t* = 330, and black: *t* = 580. Noise is defined as standard deviation over the mean. b) Experimental *y*_2_ data. c) Experimental *y*_1*r*_ data. d) Normalized experimental *y*_2_ data. Each curve is normalized by its maximum mean value in panel b. e) *y*_2_ data generated by a computational model of the circuit with slow fluctuations in protein synthesis rates. f) *y*_1*r*_ data generated by a computational model of the circuit with slow fluctuations in protein synthesis rates. g) Normalized *y*_2_ data generated by a computational model of the circuit with slow fluctuations in protein synthesis rates. Each curve is normalized by its maximum mean value in panel e.

Next, we sought to probe the major determinants of mutual information for a simple synthetic transcriptional circuit (Fig 3a). In this circuit, a constitutively expressed transcription factor $Y_{1}^{*}$ interacts with a small molecule *X*_+_, leading to the activation of the transcription factor. The active transcription factor *Y*_1_ translocates into the nucleus and activates expression of a gene *Y*_2_.

In our implementation, $Y_{1}^{*}$ is an estradiol (input *X*_+_) responsive chimeric transcription regulator (TR) consisting of three fused elements: an activation domain (from MSN2), a lipid-binding domain (from the human estradiol receptor, hER-LBD), and a DNA binding domain (from GAL4). When estradiol binds to the LBD, the activated TR *Y*_1_ translocates to the nucleus and controls the expression of promoters containing Gal4-binding-sites. Therefore, the protein *Y*_2_ (in this case a fluorescent protein) is produced from a Gal4-responsive GAL10 promoter (See Materials and Methods for more details). At the same time, $Y_{1}^{*}$ is produced from an altered version of the promoter of the alcohol dehydrogenase 1 gene (ADH1). We constructed two strains for measurement purposes. Strain 1 contains the circuit in addition to two copies of the GAL10 promoter, one driving YFP and the other driving mCherry. Strain 2 contains the circuit, but this time with two copies of the ADH1 promoter, one driving the production of $Y_{1}^{*}$ and the other driving the production of YFP (which we will refer to as *Y*_1*r*_). The same strain also contains a GAL10 promoter driving the production of the mCherry (*Y*_2_) protein. These strains were useful for two reasons. First, we wanted to establish how mutual information computations depend on the ability to simultaneously measure different quantities in a circuit (e.g. *Y*_1_ and *Y*_2_ versus *Y*_2_ alone). Given that this necessitates the use of two fluorescent proteins, in this case YFP and mCherry, we wanted to ascertain that the results we obtain are qualitatively independent of the choice of fluorophores, given that mCherry has lower dynamic range than YFP with higher background fluorescence and hence increased noise at low concentrations.

For each strain, we subjected 12 exponentially growing populations (wells) of cells cultured in non-repressive media to input concentrations of estradiol (*x*_+_) log-sampled between 0 and 100 nM. The 12 measurement points sufficiently sampled the dose response relationships. The number of cells measured from each well was greater than 3000, ensuring good statistics for approximating the MI [14]. All cultures were started from zero estradiol concentrations. Samples were taken at t = 0, 65, 165, 330 and 580 minutes. Fig 3b shows the time-dependent dose-response relationships of estradiol versus *y*_2_ (in this case YFP, strain 1) for these timepoints, where fluorescence values were normalized with respect to side scatter in order to minimize the effects of cellular volume and shape dependent differences.

The dose-response relationships of *y*_2_ normalized by their respective maximum mean values (Fig 3d) exhibit an interesting trend: for the last 3 time points, the traces for the mean and variability are very similar to each other. The only outlier to this trend is the time point at *t* = 65 minutes after stimulation (Fig 3d). For this timepoint, fluorescence is weak and strongly overlaps with autofluorescence and folding delays, and therefore the true signal cannot be accurately estimated. Autofluorescence and folding delay also contributes, albeit less dramatically, to the measurement at the *t* = 165 minutes timepoint (Fig 3d). The mCherry measurements (strain 1 or strain 2) generated the same trend (S1a and S1b Fig) albeit with a noisier outcome than YFP due to the limited dynamic range of mCherry. As a consequence, the *y*_2_ (YFP) and *y*_1*r*_ (YFP) experimental measurements from the two strains can be used in combination for comparison of modeling with data. The fact that variability in the *y*_2_ data irrespective of the fluorescent protein does not decrease with increasing mean values suggests that dominant fluctuations are unlikely to be intrinsic to the pathway.

Fig 3c plots measurements of *y*_1*r*_ (YFP, strain 2). Unexpectedly, despite the common assumption that the ADH1 promoter has constitutive and constant expression, we found that it exhibits a modest dependence on estradiol. We do not know the root of this dependence, but it is likely to reflect the influence of the circuit itself on the metabolic state of the cell, hence affecting ADH1 promoter activity. Overall, the growth rate of these strains is independent of estradiol for concentrations under 100 nM over the duration of the experiment (S1c and S1d Fig), and therefore this effect can be compensated for in the mutual information calculations. It is worth noting here that we are making the assumption that despite the fact that YFP (*Y*_1*r*_) and $Y_{1}^{*}$ are different proteins sharing only the same transcription rate (since both are driven by the ADH1 promoter), they share the same dominant noise characteristics. This would be the case if their intrinsic noise, which can be different, is insignificant compared to a dominant source of extrinsic noise affecting both. Next, we present data and modeling demonstrating that, indeed, noise in both *Y*_1_ and *Y*_2_ is most likely dominated by the same extrinsic global component.

Since the measured distributions are approximately gaussian for the majority of estradiol concentrations (S2 Fig) and the synthetic circuit (Fig 3a) follows the same basic chemical equations as the simple pathway we have studied in Fig 1b, we used this already established model to computationally explore different noise scenarios (see Materials and Methods for a more technical justification of the model). Specifically, we simulated the model (parameter values listed in Table 2) with both intrinsic variability and added global extrinsic parameter variability as sources of stochasticity. The data we collected are in fluorescent units, therefore we set our model to arbitrarily yield maximum *y*_2_ protein expression levels of about 2500 molecules, likely an underestimation of the actual system. However, this choice constitutes a scaling factor and does not affect any of our results. We also accounted for the estradiol dependence of ADH1 (Fig 3c) by adding to the model a term depicting the modest estradiol dependent repression of this promoter.

**Table 2:** Parameters for model of synthetic circuit (global model).

parameter:	*β*_*m*_1__ (mol s^−1^)	*γ*_*m*_1__ (s^−1^)	$\beta_{y_{1}^{*}}$ (s^−1^)	$\gamma_{y_{1}^{*}}=\gamma_{y_{1}}$	*θ*_*x*_	*θ*_*y*_1__	*n*_1_	*y*_1_0__	$\beta_{m_{2}}^{*}$
value:	.667	.00067	.0000139	.0000463	.002	1.5	1.5	85	0
parameter:	*β*_*m*_2__	*γ*_*m*_2__	*β*_*y*_2__	*γ*_*y*_2__ = *γ*_*y*_1*r*__	*α*_*x*_	*x*_*e*_ (mol)	*n*_*x*_
value:	.083	.00083	.0014	.0000463	.35	45	2

For global parameter variability, we again chose to focus on the parameters affecting protein expression. We potentially could model the global parameter variability with cell-to-cell heterogeneity in the protein degradation rates. However, given that our experimental data does not measure the expression of genes involved in either of these processes, i.e. no way to experimentally distinguish the source(s) of global parameter variability, we chose to model global variability in the protein creation rates. Following the same procedure as in the previous section, we added a stochastic global parameter, *G*, which affects the protein creation rates for *Y*_1_* and *Y*_2_ such that ${\hat{\beta }}_{y_{1}^{*}}=\beta_{y_{1}^{*}}G/\bar{G}$ and ${\hat{\beta }}_{y_{2}}=\beta_{y_{2}}G/\bar{G}$. The noise in the experimental *Y*_1*r*_ data is approximately .155, therefore modeling *Y*_1_ and *Y*_1*r*_ using Poissonian statistics sets the mean of the global noise variable to $\bar{G}\approx$ 42. We first assumed that global parameter fluctuations are slow relative to the circuit timescales (*γ*_*g*_ = 3 × 10^−6^). Simulating the model with this slow global source of fluctuations (SGF model, (Fig 3e–3g)) generated profiles for normalized *y*_2_ (Fig 3g) that recapitulated the highly similar variance envelopes of the experimental time-dependent dose responses (Fig 3d). This behavior was a characteristic feature of the model for any *γ*_*g*_ < = 3 × 10^−6^. By contrast, as the global fluctuating variable assumes a faster timescale (*γ*_*g*_ > 3 × 10^−6^), the variability envelopes in the normalized time-dependent dose response of *y*_2_ started to diverge from each other (S3a and S3b Fig, *γ*_*g*_ = 3 × 10^−4^). As expected, the system modeled with intrinsic variability only ($\bar{G}=\infty$, parameter values listed in Table 3) shows a normalized time-dependent dose response in which variability decreases as a function of time as the protein levels increase (S3c and S3d Fig). Given the data in Fig 3b, if the fluctuations were purely intrinsic, the ratio of the standard deviation to the mean between times 165 and 580 minutes would decrease by a factor of approximately 1.7. This is a change we should be able to detect in our data since for the number of cells sampled, the error in estimating the means and standard deviations in the dose response relationships are .5 percent and 2 percent, respectively. However, as previously discussed, the experimental data shows that this ratio is relatively invariant for the last 3 time points (Fig 3d, inset) while increasing for the both the fast global fluctuations model (S3b Fig, inset) and the intrinsic variability model (S3d Fig, inset). Our argument is further strengthened by the fact that in order to capture the noise observed in *y*_1*r*_ with the intrinsic variability model for the first timepoint, we had to set the *y*_1*r*_ mean copy number in the model to an unrealistically low value for a strong promoter such as ADH1 (approximately 40 proteins), further indicating that variability is unlikely to be intrinsic. The results for the SGF model for *γ*_*g*_ < = 3 × 10^−6^ are not an artifact of the estradiol dependence of ADH1 since an SGF model without this effect yields indistinguishable results (S3e and S3f Fig). We therefore conclude that the dominant source of variability in this synthetic circuit is likely to be due to a globally slow fluctuating variable. This is consistent with previous results, which also indicated that global parameters play a dominant role in cell to cell variability and that these parameters exhibit fluctuations at a slower timescale than fluctuations of processes involved in gene expression [4].

**Table 3:** Parameters for model of synthetic circuit (intrinsic model).

parameter:	*β*_*m*_1__	*γ*_*m*_1__	$\beta_{y_{1}^{*}}$	$\gamma_{y_{1}^{*}}=\gamma_{y_{1}}$	*θ*_*x*_	*θ*_*y*_1__	*n*_1_	*y*_1_0__	$\beta_{m_{2}}^{*}$	*β*_*m*_2__	*γ*_*m*_2__
value:	.667	.00067	.0000019	.0000463	.002	1.5	1.5	1	0	.083	.00083
parameter:	*β*_*y*_2__	*γ*_*y*_2__ = *γ*_*y*_1*r*__	*α*_*x*_	*x*_*e*_	*n*_*x*_
value:	.0014	.0000463	.35	45	2

**Fig 4:**
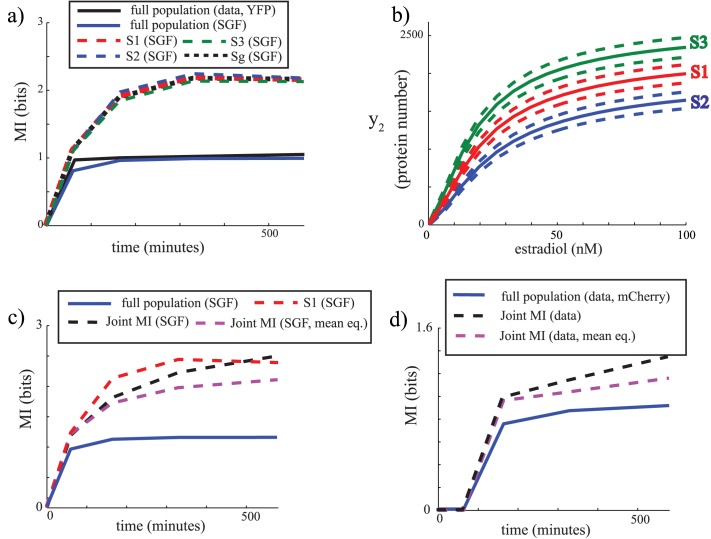
Mutual information modeling predictions and experimental measurements of the transcriptional synthetic circuit. a) Mutual information *I*(*x*_+_; *y*_2_, *t*) from experimental data (black solid, YFP in Strain 1), *I*(*x*_+_; *y*_2_, *t*) from the SGF model (blue solid), and *I*(*x*_+_; *y*_2_, *t*∣*Si*) from the SGF model conditioned on S1 (red dashed), S2 (blue dashed), S3 (green dashed), and Sg (black dashed). b) Dose response of *y*_2_ as a function of estradiol at time 580 minutes for the SGF model with initial condition S1, S2, and S3. c) Mutual information *I*(*x*_+_;[*y*_1*r*_ *y*_2_], *t*) based on joint measurement of *y*_1*r*_ and *y*_2_ computed for the SGF model (black dashed), same calculations for equalized mean of *y*_1*r*_ (magenta dashed). Also shown for comparison are the mutual informations *I*(*x*_+_; *y*_2_, *t*) (SGF, blue solid) and *I*(*x*_+_; *y*_2_, *t*∣*S*1) (SGF, red dashed). d) Measured joint mutual information *I*(*x*_+_;[*y*_1*r*_ *y*_2_], *t*) (black dashed) including the mean equalized case (magenta dashed). The measured mutual information *I*(*x*_+_; *y*_2_, *t*) (mCherry, Strain 2) is also shown for comparison (blue solid).

In terms of mutual information, the fact that the normalized time-dependent dose responses coincide in terms of their variability (Fig 3d) implies that the experimentally computed mutual information *I*(*x*_+_, *y*_2_, *t*) at *t* = 165, 330 and 580 minutes should be similar. This is indeed the case (Fig 4a, solid black). Importantly, *I*(*x*_+_, *y*_2_, *t*) peaks and reaches a plateau at approximately 1 bit, at an earlier time than when *y*_2_ reaches its steady-sate. This further lends credence to the idea that the variability in the population is dominated by global parameter variability. Gratifyingly, the model with ‘slow’ global parameter fluctuations (with *γ*_*g*_ = 3 × 10^−6^) also captures the time-dependent mutual information seen in the data without any further parameter tuning (Fig 4a, solid blue).

Since slow global fluctuations seem to dominate in this circuit, our analysis above indicates that the population mutual information might be under-estimating the fidelity of a single cell. To illustrate this point, we used the model to computationally isolate and compute the mutual information *I*(*x*_+_; *y*_2_, *t*∣*Si*) for single cells S1, S2 and S3 as defined in the computational example above. These calculations yield a substantially higher MI value than the population MI for the time span simulated (Fig 4a). Evidently, and as explained above, the MI for S1, S2, or S3 will eventually converge back to the whole population MI, but here it will do so on a much slower timescale than that of the system. For example, the dose response and distribution of *y*_2_ at time 580 minutes when the system is started from S1, S2 and S3 (Fig 4b) still shows tighter variability than that of the full population. Allowing for intrinsic variability in the initial conditions, i.e. starting with cells with $G=\bar{G}$ at time zero (state *S*_*g*_) yields a similar MI value to that of *I*(*x*_+_; *y*_2_, *t*∣*S*1) for *γ*_*g*_ = 3 × 10^−6^ (Fig 4a).

Finally, we explored how simultaneous measurement of *y*_1*r*_ and *y*_2_ affects mutual information calculations. Calculations using the model indicate that as expected, knowledge of *y*_1*r*_ improves the estimate of mutual information. For a slow globally fluctuating variable (*γ*_*g*_ = 3 × 10^−6^), the joint mutual information *I*(*x*_+_; [*y*_1*r*_ *y*_2_], *t*) is larger than *I*(*x*_+_; *y*_2_, *t*). It can be shown that *I*(*x*_+_; [*y*_1*r*_ *y*_2_], *t*) = *I*(*x*_+_; *y*_1*r*_, *t*) + E[*I*(*x*_+_; *y*_2_, *t*∣*y*_1*r*_)] where E[*I*(*x*_+_; *y*_2_, *t*∣*y*_1*r*_)] is the expected value of *I*(*x*_+_; *y*_2_, *t*∣*y*_1*r*_). Since the influence of estradiol on *y*_1*r*_ adds (albeit very slightly (Fig 3c (data) and Fig 3f (model)) to the mutual information, i.e. *I*(*x*_+_; *y*_1*r*_, *t*) > 0, we normalized for this effect. To do so, at a given time, we set the *y*_1*r*_ mean at each estradiol value to the value of the *y*_1*r*_ mean at zero estradiol while adjusting the variance to preserve the noise in *y*_1*r*_ at each estradiol value. Importantly, this operation does not affect correlation between *y*_2_ and *y*_1*r*_ at each estradiol value, but enforces *I*(*x*_+_; *y*_1*r*_, *t*) = 0. We confirm that this does not change our conclusions that knowledge of *y*_1*r*_ improves the estimate of mutual information (compare Fig 4c blue, dashed black and dashed magenta).

For comparison, we can carry out mutual information from the data obtained using Strain 2 in which both *y*_1*r*_ and *y*_2_ are measured. In this strain, *y*_2_ is the fluorescent protein mCherry which has a limited dynamic range. Importantly, at the highest estradiol values and peak mCherry signal (time 580 minutes), the measured correlation between *y*_1*r*_ and *y*_2_ (greater than .79) is less than ten percent below the model predictions. Even at these signal levels the noise in the mCherry signal still deteriorates the correlation. For decreasing values of estradiol the correlations become increasingly inaccurate. Therefore, the values of the MI cannot be quantitatively compared to the model which was fitted to YFP data. However, the qualitative trend of increased MI due to measurement of *y*_1*r*_ relative to computing the MI with no knowledge of *y*_1*r*_ should also hold. This is indeed seen to be the case (Fig 4d). This insight is in agreement with recent work [19] that studied mutual information in the RAS/ERK pathway. Nuclear ERK (*erk*_*nuc*_) was used as a readout of pathway information transmission. The MI at time *t* between this readout and the input *x* was conditioned for single cell ERK levels, using measurement of total ERK (*erk*_*tot*_). It was also shown that *I*(*x*; [*erk*_*tot*_ *erk*_*nuc*_], *t*) is greater than *I*(*x*; *erk*_*nuc*_, *t*). Therefore, simultaneous measurements of different cellular variables improve estimates of mutual information capabilities of single cells.

## Discussion

In this work, we illustrated how variability in initial conditions across a population, as well as slow-fluctuating extrinsic (global) variables can generate low values for the population mutual information in response to an input. We also demonstrated that when subpopulations of cells that have similar parameters or initial states are isolated, their mutual information values are transiently much higher than those of the whole population. These findings are important in light of the fact that many previous studies have found that extrinsic variability is a substantial contributor to pathway fluctuations. Indeed, our own experimental data using a synthetic circuit also implicated extrinsic fluctuations as a major source of variability. As a result, cells in a population cannot be considered to be the same noisy channel for mutual information calculations. Rather, each cell is a different noisy channel possessing its own parameters. Recent work [20] using light-inducible input signals [21, 22] to a mammalian RAS/MAPK pathway observed that different isogenic single cells have quantitatively different dose-response relationships. Interestingly, for the RAS/MAPK mammalian system, the dose-repsonse relationships were repeatable for hours within a given single cell [20], suggesting slow global parameters that affect that pathway for that duration.

A direct assessment of mutual information requires repeated time-resolved measurements in single cells. Another strategy to better approximate mutual information is to simultaneously measure a large number of interconnected variables, including global states. This might be increasingly feasible with breakthrough technologies such as mass-cytometry (a.k.a. CyTOF) [23] as well as improvements in fluorescent reporter technologies. In the mean time, however, we have demonstrated that computational modeling, especially with respect to the patterns of time-dependent variability, can generate valuable insights into whether intrinsic or extrinsic fluctuations dominate variability in a circuit. These results produce a more accurate quantification of mutual information, and therefore promise to generate a more realistic assessment of signaling fidelity in cellular circuits.

Our results and those from [20] support a view in which individual cells have distinct transfer functions over relevant signaling timescales, and have superior signaling fidelity (> 1.5 bits) than estimated from pooled measurements of a population. From this perspective, it could be the case that a diversity of high fidelity but different single cell signaling transfer functions across the population is a beneficial trait. However, some situations might arise where variability in population signal transmission capacity is not desirable. In this case, cells might use strategies such as negative feedback to constrain this variability. In either case, cells might also capitalize on the integration of signals from many pathways that respond to a given input(s) in order to generate a desired population response. In this view, each such pathway will add to the mutual information of the desired cellular output (e.g. level or activity of a transcription factor), allowing the population to further circumvent in this way any information fidelity bottleneck. Researchers of the subject are likely to encounter both situations, and perhaps a revised form of population mutual information might be needed to quantify these effects, along with the formulation of new information theoretic metrics. As an example, for any given input *x*(*t*), the mutual information $I(y_{2};y_{1r}^{*},t|x(t))$ gives us a sense of the diversity (or spread) in responses in *y*_2_ given the cell-to-cell variability encoded in $y_{1}^{*}$. S4 Fig shows the results of this metric applied to the simple signaling cascade (Fig 1b) for different input step function amplitudes *x*_+_ and for different times. We envision these kind of metrics to reflect the different subpopulations with similar parameters within a given population and to serve as a potential tool to quantify how cell-to-cell variability across a population might change in structure due to various time-dependent inputs.

Finally, most studies to date have focused on variability in populations of non-communicating cells. Information fidelity in cells that communicate, for example through quorum sensing for bacterial communities or cell-to-cell mechanical coupling for tissues, is still largely unstudied. How cell-to-cell communication modulates global variability and variability in initial conditions across a population, and hence mutual information of cellular pathways, is a topic that should be explored in order to determine whether and when multicellularity offers a beneficial strategy in terms of signaling fidelity.

## Materials and Methods

### Approximating mutual information with N experiments

Because we are using a finite number of experiments, the input distribution *p*_*u*_(*x*_+_) is sampled with *N* discrete points. In practice, these points are spaced to accurately sample the input-output transfer function *p*(*y*, *t*∣*x*_+_) for *x*_+_ ranging from 0 to *X*_*max*_. The time-dependent mutual information is then calculated with this data. For values of *x*_+_ between the sampled values, *p*(*y*, *t*∣*x*_+_) is approximated by linearly interpolating the moments of the adjacent sampled distributions. Since the distributions generated by systems in this paper are approximately gaussian (and approximately negative binomial at very low *x*_+_ for the synthetic circuit data), only the means and covariances are required. A larger number of experiments (*N*) generates a more accurate approximation of mutual information. However, we observed that convergence to accurate MI values does not increase monotonically with *N* for the logarithmic sampling of the doses response that we have adopted. Rather, convergence proceeds exponentially, followed by marginal gains in accuracy as *N* increases. Therefore, for every *N*, we examine the last three sample number values, *N*, *N*−1 and *N*−2. Given their measured convergence rates, we can extrapolate an upper bound on the MI at an infinite number samples. We choose *N* whose calculated MI at *N* is within 1 percent of the extrapolated upper bound.

### Chemical equations for the simple *in silico* network

The chemical equations for the circuit in Fig 1b are

$$\begin{array}{c} \emptyset \;\overset{\beta_{y_{1}^{*}}}{⟶}\;Y_{1}^{*}\;\overset{\gamma_{y_{1}^{*}}Y_{1}^{*}}{⟶}\;\emptyset \end{array}$$ (2a)

$$\begin{array}{c} X+Y_{1}^{*}\;\;\overset{\theta_{x}XY_{1}^{*}}{⟶}\;\;Y_{1}\;\;\overset{\theta_{y_{1}}Y_{1}}{⟶}\;\;Y_{1}^{*} \end{array}$$ (2b)

$$\begin{array}{c} Y_{1}\;\;\overset{\gamma_{y_{1}}Y_{1}}{⟶}\;\;\emptyset \end{array}$$ (2c)

$$\begin{array}{c} \emptyset \;\;\overset{f_{1}\left(y_{1}\right)}{⟶}\;\;M_{y_{2}}\overset{\gamma_{m_{2}}M_{y_{2}}}{⟶}\;\;\emptyset \end{array}$$ (2d)

$$\begin{array}{c} \emptyset \;\;\overset{\beta_{y_{2}}M_{y_{2}}}{⟶}\;\;Y_{2}\overset{\gamma_{y_{2}}Y_{2}}{⟶}\;\;\emptyset \end{array}$$ (2e)

where $f_{1}(y_{1})=\beta_{m_{2}}^{*}+\frac{\beta_{m_{2}}y_{1}^{n_{1}}}{y_{1}^{n_{1}}+y_{1_{0}}^{n_{1}}}$. The propensities of the reactions appear above the reaction arrows. The system is a simple cascade of reactions where the input *X* activates *Y*_1_, and subsequently the *Y*_1_-dependent transcription of *Y*_2_. The parameter values are tabulated in Table 1.

Here the mean total number of *Y*_1_ molecules, active and inactive, is $\beta_{y_{1}^{*}}/\gamma_{y_{1}^{*}}=1500$. The max mean numbers for *Y*_2_ mRNA and *Y*_2_ protein are *β*_*m*_2__/*γ*_*m*_2__ = 200 and $\frac{\beta_{m_{2}}\beta_{y_{2}}}{\gamma_{m_{2}}\beta_{y_{2}}}=2000$, respectively. This system has only a single stationary solution. This allows us to approximate and efficiently calculate the master equation with a local affine assumption using the first two moments Eqs (6) and (7) taken from [24].

#### Inclusion of global parameter variability within chemical equations

In this work, we have assumed that the stochastic global parameter, *G*, manifests itself in variation in translation rates. To incorporate *G* we define our new protein creations rates ${\hat{\beta }}_{y_{1}^{*}}=\beta_{y_{1}^{*}}G/\bar{G}$ and ${\hat{\beta }}_{y_{2}}=\beta_{y_{2}}G/\bar{G}$. Here, $\beta_{y_{1}^{*}}$ and *β*_*y*_2__ are the nominal values in Eq (2). For simplicity we define *G* to follow a memoryless birth/death process through the reaction equations

$$\begin{array}{c} \emptyset \;\;\overset{\beta_{g}}{⟶}\;\;G\;\;\overset{\gamma_{g}G}{⟶}\;\;\emptyset \end{array}$$ (3)

where $\bar{G}=\beta_{g}/\gamma_{g}$ and the noise in *G*, *η*_*g*_, is given as the standard deviation over the mean: $\eta_{g}=1/\sqrt{\bar{G}}$.

#### Chemical equations for the synthetic circuit

The chemical equations for the synthetic circuit in Fig 3a are the same as the simple circuit except that the production of $Y_{1}^{*}$ now involves an mRNA step, which does not directly affect any of our results. Also, we have added a YFP reporter of $Y_{1}^{*}$ that has a half-life of 6 hours which we set the transcription factor itself to be the same. We define the estradiol dependence in $Y_{1}^{*}$ mRNA as $\beta_{m_{1}^{*}}(x)=\beta_{m_{1}}(1-\alpha_{x})+\alpha_{x}x_{e}^{n_{x}}/(x_{e}^{n_{x}}+x^{n_{x}})$ The parameters for the circuit are given in Table 2 (global fluctuation model) and Table 3 (intrinsic fluctuation model).

### Computation of first two moments using affine assumption

The formulation that we assume in our model and data consists of a system of well-stirred chemical reactions with *N* molecular species. For some environmental input *X*(*t*), we define the pathway state *Y*(*t*) to denote the vector whose integer elements *Y*_*i*_(*t*) are the number of molecules of the *i*th species at time *t*. If there are *M* elementary chemical reactions that can occur among these *N* species, then we associate with each reaction *r*_*j*_ (*j* = 1, …, *M*) a non-negative *propensity function* defined such that *a*_*j*_(*Y*(*t*)) *τ*+*o*(*τ*) is the probability that reaction *r*_*j*_ will happen in the next small time interval [*t*, *t*+*τ*], as *τ* → 0. The polynomial form of the propensities *a*_*j*_(*y*) may be derived from fundamental principles under certain assumptions [25]. The occurrence of a reaction *r*_*j*_ leads to a change of *ν*_*j*_ ∈ **Z** ^*N*^ (the set of nonnegative integers) for the state *Y*. *ν*_*j*_ is therefore a stoichiometric vector that reflects the integer change in reactant species due to a reaction *r*_*j*_.

This set of well-stirred chemical reactions can be represented by the joint probability density function *P*(*y*, *t*∣ *X*(*t*)) which describes the probability of the system being in state *y* at time *t*, given the environmental signal *X*(*t*). The evolution of *P*(*y*, *t*∣*X*(*t*)) is given by

$$\begin{array}{c} \frac{\partial P(y,t|X(t))}{\partial t}=\sum_{j=1}^{M}[a_{j}\left(y-\nu_{j}\right)P\left(y-\nu_{j},t|X\left(t\right)\right)-a_{j}\left(y\right)P\left(y,t|X\left(t\right)\right)] \end{array}$$ (4)

Eq (4) is the so-called chemical master equation (CME)[26, 27].

To approximate the CME with moment equations, we approximate the propensity function *a*_*j*_(*y*) with a locally affine Taylor series expansion [24] about the mean of the distribution, *z*(*t*), to get

$$\begin{array}{c} a_{j}\left(y\right)\approx a_{j}\left(z\left(t\right)\right)+\sum_{i=1}^{N}\frac{\partial a_{j}\left(y\right)}{\partial y_{i}}{|}_{y=z(t)}\left[y_{i}-z_{i}\left(t\right)\right] \end{array}$$ (5)

From the time dependent mean equation for the *k*th species is

$$\begin{array}{c} \frac{\partial z_{k}\left(t\right)}{\partial t}=\sum_{j=1}^{K}\nu_{jk}a_{j}\left(z\left(t\right)\right) \end{array}$$ (6)

and the time dependent covariance equation for the *k*th and *k*′th species is

$$\begin{array}{ccc} \frac{\partial C_{kk^{\prime }}\left(t\right)}{\partial t} & = & \sum_{j=1}^{K}(\nu_{jk}\sum_{i}\frac{\partial a_{j}\left(y\right)}{\partial y_{i}}{{|}_{y=z(t)}C_{ik^{\prime }}+\nu_{jk^{\prime }}\sum_{i^{\prime }}\frac{\partial a_{j}\left(y\right)}{\partial y_{i^{\prime }}}|}_{y=z(t)}C_{ki^{\prime }}\\ & & +\nu_{jk}\nu_{jk^{\prime }}a_{j}\left(z\left(t\right)\right)) \end{array}$$ (7)

The calculation of the mutual information requires probability distributions. Given that we solve the first two moments, we constrain our distributions to be either a negative binomial distribution or a normal distribution. For cases when $\mu_{k}<3\sqrt{(}C_{kk})$, we apply the negative binomial distribution since it only requires the first two moments and is non-negative. The negative binomial is very close to a normal distribution for $\mu_{k}>3*\sqrt{(}C_{kk})$ and we therefore apply the normal distribution in these regions. The value of 3 used is heuristic, but the tail of the normal distribution at negative values is negligible at this point. For linear transcriptional systems, the negative binomial is a natural steady state solution [28] which was our motivation for applying it. Importantly, our data never violated any constraints required by the negative binomial distribution, for example, *μ*_*k*_ ≤ *C*_*kk*_. Note that the negative binomial distribution is only required for our modeling of the synthetic circuit data. Our theoretical example in the first half of the paper has large enough basal levels at zero input which always keeps it in the normal distribution regime. As a demonstration of the validity of the moment approach, S5 Fig shows very good agreement in the distributions derived from stochastic simulations (SSA) and the moment equations for the synthetic circuit model (*γ*_*g*_ = 3 × 10^−6^, initial condition S1).

### Synthetic circuit constructs

#### Strains

All plasmids used in this study were derived from a set of yeast single integration vectors containing selectable markers and targeting sequences for the LEU2, HIS3, TRP1 and URA3 loci. These vectors were linearized by digestion with PmeI and transformed using standard yeast transformation techniques. All strains were derived from haploid W303a and were deleted for GAL4 to eliminate competition between the endogenous Gal4p and the previously described estradiol-inducible Gal4 chimera (Gal4DBD-ER) for binding to the GAL10 promoter [29]. The sequences for the ADH1 and GAL10 promoters were 658 and 646 bp upstream from the start codons for these genes, respectively. The genotypes for these strains are listed in Table 4.

**Table 4:** Strains used in this study.

Strain name	Genotype
S-1	W303 MATa leu2::LEU2-P_*GAL10*_Venus, trp1, ura3::URA3-P_*ADH1*_Gal4DBD-ER, ade2::ADE2, his3::HIS3-P_*GAL10*_mCherry, GAL4::HPH
S-2	W303 MATa leu2, trp1::TRP1-P_*ADH1*_Venus, ura3::URA3-P_*ADH1*_Gal4DBD-ER, ade2::ADE2, his3::HIS3-P_*GAL10*_mCherry, GAL4::HPH

#### Growth conditions and flow cytometry

Cells were grown in YPD at 30°C. Prior to the experiment, cells were grown and maintained in exponential phase (optical density <.15) for approximately twenty-four hours in the absence of estradiol and then diluted to an optical density (OD600) of 0.05 at the beginning of the experiment and periodically diluted to stay under 0.15. Using a deep-well 96-well plate (Thermo), estradiol concentrations decreased from columns one to eleven in logarithmically spaced points according to the following equation: 100(2/3)^*c*−1^ nM where c denotes the column number. Well 12 did not receive estradiol. Estradiol did not change the growth rate of the cell population. Two replicates were performed. Measurements were taken at t = 0, 65, 165, 330, and 580 minutes.

Fluorescence measurements were performed on a LSRII analyzer (BD Biosciences). A blue (488 nm) laser was used to excite YFP (Venus) and a green (561 nm) laser was used to excite RFP (mCherry). Emission was detected using a 530/30-nm bandpass filter for Venus (Chroma) and a 610/20 bandpass filter for mCherry (Chroma). Greater than three thousand cells were collected for each measurement. Flow cytometry data was analyzed in MATLAB (Mathworks).

### Mutual information of multivariate measurements

Here we discuss how multi-variate MI measurements relates to MI measurements from particular initial conditions: We start with the distribution *p*(*y*_*m*_, *y*_*s*_, *t*∣*x*(*t*)) where *y*_*m*_ are the dynamic cellular pathway/network signals, *y*_*s*_ are the slowly fluctuating pathway component quantities relative to the timescale of a given experiment, and *x*(*t*) is the input signal(s). The time dependent mutual information is

$$\begin{array}{ccc} I(x\left(t\right);\left[y_{m}\;y_{s}\right],t) & = & \sum_{x(t)}\sum_{y_{s}}\sum_{y_{m}}p\left(y_{m},y_{s},t|x\left(t\right)\right)p\left(x\left(t\right)\right)\log_{2}\frac{p(y_{m},y_{s},t|x\left(t\right))}{p(y_{m},y_{s},t)}\\ & = & \sum_{x(t)}\sum_{y_{s}}\sum_{y_{m}}p\left(y_{m},t|y_{s},x\left(t\right)\right)p\left(y_{s},t|x\left(t\right)\right)p\left(x\left(t\right)\right)\log_{2}\frac{p\left(y_{m},t|y_{s},x\left(t\right)\right)p\left(y_{s},t|x\left(t\right)\right)}{p\left(y_{m},t|y_{s}\right)p\left(y_{s},t\right)} \end{array}$$ (8)

where the second line is simply a chain-rule representation. In addition to the assumption that the quantities of *y*_*s*_ are fluctuating extremely slowly, we will also impose that the quantities in *y*_*s*_ are independent of x(t). This results in *p*(*y*_*s*_, *t*∣*x*(*t*)) = *p*(*y*_*s*_, *t*) ≈ *p*(*y*_*s*_). The time dependent MI is approximated as

$$\begin{array}{ccc} I(x\left(t\right);\left[y_{m}\;y_{s}\right],t) & \approx & \sum_{x(t)}\sum_{y_{s}}\sum_{y_{m}}p\left(y_{m},t|y_{s},x\left(t\right)\right)p\left(y_{s}\right)p\left(x\left(t\right)\right)\log_{2}\frac{p\left(y_{m},t|y_{s},x\left(t\right)\right)p\left(y_{s}\right)}{p\left(y_{m},t|y_{s}\right)p\left(y_{s}\right)}\\ & = & \sum_{y_{s}}p\left(y_{s}\right)\sum_{x(t)}\sum_{y_{m}}p\left(y_{m},t|y_{s},x\left(t\right)\right)p\left(y_{s}\right)p\left(x\left(t\right)\right)\log_{2}\frac{p(y_{m},t|y_{s},x\left(t\right))}{p(y_{m},t|y_{s})}\\ & = & \sum_{y_{s}}p\left(y_{s}\right)I\left(x\left(t\right);y_{m},t|y_{s}\right)\\ & = & E[I(x\left(t\right);y_{m},t|y_{s})] \end{array}$$ (9)

Finally, we can examine the mutual information between *y*_*s*_ and *y*_*m*_ for a given input signal(s) *x*(*t*) using the formula

$$\begin{array}{ccc} I(y_{m};y_{s},t|x\left(t\right)) & = & \sum_{y_{s}}\sum_{y_{m}}p\left(y_{m},t|y_{s},x\left(t\right)\right)p\left(y_{s},t|x\left(t\right)\right)\log_{2}\frac{p(y_{m},t|y_{s},x\left(t\right))}{p(y_{m},t|x\left(t\right))} \end{array}$$ (10)

## Supporting Information

S1 Fig Comparison of *Y*_2_ mCherry measurements show that Strains 1 and 2 are equivalent in *Y*_2_ output. Strains 1 and 2 have similar growth rates that are independent of estradiol concentrations less than 100 nM. a-b) Dose-response relationships for *t* = 65 (blue), 165 (green), 330 (red), and 580 (black) minutes. The top panel is the normalized experimental *y*_2_ data. Each curve is normalized by its maximum mean value in the corresponding lower panel. The lower panel is the un-normalized experimental *y*_2_ data. a) Experimental *y*_2_ (mCherry) data for Strain 1. b) Experimental *y*_2_ (mCherry) data for Strain 2. c)-d) Single well growth curves accouting for dilution of 1/2 at *t* = 165 and 1/2 at *t* = 330 (single wells (red), 0 nM estradiol well (blue), 100 nM estradiol well (green), average (black)). The flow cytometer sample volume changed at *t* = 165 minutes, hence the jump in cell count. There was also pipetting error from the deep well plate to the sample plate at *t* = 0 and 65 minutes resulting in error in cell count across wells. For *t* = 165, 330 and 580 the pipetting error was minimized. c) Growth curves for Strain 1. d) Growth curves for Strain 2.
(EPS)

S2 Fig The majority of peak normalized dose-dependent distributions of *Y*_2_ (YFP) for estradiol doses from 2.6 to 44.4 nM are close to gaussian. The distributions presented in ascending mean values are for ascending estradiol nM values of 2.60, 5.85, 8.78, 13.2, 19.8, 29.6, and 44.4, respectively. a) Peak normalized distributions of *Y*_2_ (YFP) at 580 minutes. b) Close-up of peak normalized distributions at 580 minutes for low doses of estradiol. c) Peak normalized distributions of *Y*_2_ (YFP) at 165 minutes. d) Close-up of peak normalized distributions at 165 minutes for low doses of estradiol.
(EPS)

S3 Fig Comparison of alternative computational models for the transcriptional synthetic circuit demonstrate that only the slow global fluctuations model can recapitulate the experimental data. a)-f) Dose-response relationships for *t* = 65 (blue), 165 (green), 330 (red), and 580 (black) minutes for alternative computational models. Insets in b, d and f represent the ratio of the noise at time *t* to that at time *t* = 580 where for green: *t* = 165, red: *t* = 330, and black: *t* = 580. Noise is defined as standard deviation over the mean. a)-b) Fast global fluctuations model. a) *y*_1*r*_ data. b) Normalized *y*_2_ data. Each curve is normalized by its maximum mean value. c)-d) Intrinsic fluctuations model. c) *y*_1*r*_ data. d) Normalized *y*_2_ data. Each curve is normalized by its maximum mean value. e)-f) Slow global fluctuations model with no estradiol dependent effects on *y*_1*r*_ production. e) *y*_1*r*_ data. f) Normalized *y*_2_ data. Each curve is normalized by its maximum mean value.
(EPS)

S4 FigTime snapshots of mutual information, $I(y_{2};y_{1r}^{*},t|x(t))$.The mutual information is applied to the simple pathway model with slow global model fluctuations for *γ*_*g*_ = 3 × 10^−8^ and $\bar{G}=50$. Here *x*(*t*) is a simple step function with amplitude *x*_+_. Therefore, the range of *x*_+_ represents a range of different *x*(*t*). Times are *t* = 65 (blue), 165 (green), 330 (red), and 750 (black) minutes.(EPS)

S5 Fig Comparison of SSA and moment equations for the synthetic circuit model (*γ*_*g*_ = 3 × 10^−6^ and initial condition S1). a) Plot of dose response from moment equations at time 580 minutes. b) Plots of the distribution *p*(*y*_2_, *t* = 580∣*x*(*t*))/max(*p*(*y*_2_, *t* = 580∣*x*(*t*))) derived from the SSA and moment equations for select estradiol values up to 8.5 nM. c) Plots of the distribution *p*(*g*, *t* = 580∣*x*(*t*))/max(*p*(*g*, *t* = 580∣*x*(*t*))) derived from the SSA and moment equations. Analytical steady-state distribution shows that the moment equations and SSA results have evolved the global variable distribution *p*(*g*, *t* = 580∣*x*(*t*)) about halfway to steady state.
(EPS)
